# A Comparative Study in Microstructure and Mechanical Properties of Symmetric and Asymmetric Double-Sided FSW Joints of AA7A65

**DOI:** 10.3390/ma18030645

**Published:** 2025-01-31

**Authors:** Chen Chen, Yichao Zhu, Zhiping He, Hongjian Lu, Weifeng Xu, Tengfei Cui, Wenjing Liu, Chenyang Qiu, Zhanping Gao

**Affiliations:** 1 China Helicopter Research and Development Institute, Jingdezhen 333001, China; hezp@avic.com (Z.H.);; 2 School of Materials Science and Engineering, Northwestern Polytechnical University, Xi’an 710072, China; zhuyc@avic.com (Y.Z.); xwf1982@nwpu.edu.cn (W.X.)

**Keywords:** asymmetric double-sided friction stir welding, aluminum alloy, microstructure, mechanical properties

## Abstract

Double-sided friction stir welding (DS-FSW) demands a low requirement of the welding tool and equipment and can lower the heat input, showing advantages in joining thick-plate joints. However, the intrinsic twice thermal cycle inevitably leads to the twice grain growth and softening, troubling the performance of the joints. To alleviate this phenomenon, this work proposed an asymmetric DS-FSW (Asy-DS-FSW) in which the first weld and second weld are obtained via a large tool and a small tool, respectively. The results suggest that the Asy-DS-FSW effectively refines the grains and inhibits twice-grain growth in the 27 mm thick AA7A65 joints. The hardness of Asy-DS-FSW is higher and more homogeneously distributed than the conventional symmetric DS-FSW (Sym-DS-FSW). The ultimate tensile strength of the slices is enhanced by 0.5–11%, and the eased strain localization can be achieved by the Asy-DS-FSW, compared with the Sym-DS-FSW. This work offers valuable references to the high-quality joining of thick-plate aluminum alloys in aerospace.

## 1. Introduction

The Al-Zn-Mg-Cu (AA7×××) aluminum alloys that own superior strength to the other series of aluminum alloys show great advantages when they are applied in the aircraft [1,2,3,4]. Lowering the structural weight is beneficial to energy saving and cost reduction. Applying the welding methods to replace the riveting is believed to be effective. However, metallurgical defects like porosity, oxidation, and cracking are prone to form in these AA7××× high-strength aluminum alloy joints during fusion welding. Benefiting from the high energy efficiency, low defect tendency, and relatively superior mechanical properties, friction stir welding (FSW), a solid-state welding technology, is promising for realizing the high-quality joining of aluminum alloy components in aircraft manufacturing.

Al-Zn-Mg-Cu alloys are widely used for large-scale thick-section structures like fuselage frames and bulkheads, etc. [1,4]. However, it is difficult for the thick-plate FSW joints (>10 mm) to obtain the joining efficiency as high as the thin-plate FSW joints (<10 mm). The joining efficiency (ultimate tensile strength ratio of the joint to BM) of the thick-plate joints is approximately 70%, while that of the thin-plate joints is always higher than 80% [5,6,7,8,9]. The widespread of FSW structures in the aircrafts is thus limited. The inferior mechanical properties of thick-plate joints are mainly led by the typical characteristics of the FSW. There is generally a temperature gradient along the thickness direction of FSW joints. This phenomenon may become more prominent as the thickness of plates increases. It is widely accepted that the higher welding heat input may make the heat-affected zone in high-strength aluminum alloy FSW joints softer owing to more severe precipitates coarsening and dissolution [10,11,12,13]. However, the lower welding heat input may make it easier to generate defects like kissing bonding, loosening, voids, etc. [14,15]. Besides, the heterogeneous distribution of the thermal-mechanical coupling effect will result in microstructural heterogeneity along the common transverse direction and especially the thickness direction in thick-plate joint. The degree of strain localization may further increase and accelerate the failure of the joint.

Many efforts have been made to realize the no-defect FSW under the lower welding heat input and thereby obtain the joints with superior performance [16,17,18,19,20,21,22,23,24,25,26,27,28]. Especially for the thick-plate FSW, turning the single-pass welding into two-pass welding is believed as a useful method to lower the welding heat generation per pass. There are many two-pass FSW technologies like the bobbin tool FSW (BB-FSW) [7,24,25,26], the double-side FSW (DS-FSW) [6,13,29], and the synergistic double-side FSW (SDS-FSW) [27]. However, it is reported that the joining efficiency of the BB-FSW joint is only 32%, and joints fail in the form of intergranular brittle fracture in the weld nugget zone (WNZ) with an elongation of no more than 0.4% [7], inferring the non-feasibility of the BB-FSW in thick-plate joints. Besides, to control the deformation of the profiles to be welded and avoid the collision of two simultaneous rotating pins, the sum of the length of the pin applied in the SDS-FSW is smaller than the thickness of the plate, which eventually leaves an unwelded area in the center of weld [27]. The unwelded area will act as the crack initiation site under the external and promote the failure of the components [27]. The existence of the unwelded area is unacceptable in actual production. Further, the SDS-FSW needs two rotating spindles, which highly demands special equipment. Compared with the BB-FSW and the SDS-FSW, the DS-FSW demands fewer special welding tools or equipment, and the welding process can be more flexible, showing technical advantages. Noteworthy, the welding tools applied for the two welds of DS-FSW in existing works are always the same, which is the symmetric DS-FSW (Sym-DS-FSW) process. It is widely known that the welding heat input in the latter welds will surely take action at the former welds during the multi-pass welding [30,31,32]. The performance of the high-strength aluminum alloys whose microstructure is sensitive to elevated temperature is further significantly affected. Xu et al. [6], Zou et al. [27], and Yang et al. [29] have observed a prominent softening zone in the middle of the DS-FSW joint induced by the twice heat input. Applying another smaller welding tool at the second weld may be a promising method to alleviate this effect, i.e., using the asymmetric DS-FSW (Asy-DS-FSW) process. This is because the Asy-DS-FSW may generate less heat at the second weld, and the microstructure in the first weld will be thus less affected compared with the Sym-DS-FSW. It is reasonably expected that the Asy-DS-FSW can not only own the advantages similar to the Sym-DS-FSW but also further improve the performance of the thick-plate joints. Nevertheless, the study on the effect of Asy-DS-FSW on the microstructure and mechanical properties of thick-plate is limited. A detailed study on it may help the manufacture of thick-plate joints in aircraft.

Therefore, in this work, the DS-FSW was carried out with the AA7A65 in the form of both symmetry and asymmetry. The AA7A65 is a new aluminum alloy with a high Zn/Mg ratio, and it has good strength, toughness, and corrosion resistance [33]. It is a promising structural aluminum alloy in aircraft. The microstructure and the mechanical properties of the two joints were studied and compared, which can deepen the understanding of the Asy-DS-FSW and provide useful guidance for the follow-up study and the actual aircraft production.

## 2. Materials and Experiments

**Figure 1:**
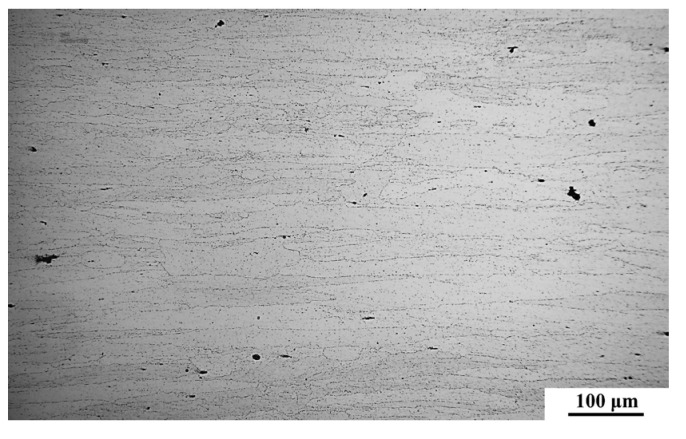
The OM image of the AA7A65 BM.

**Figure 2:**
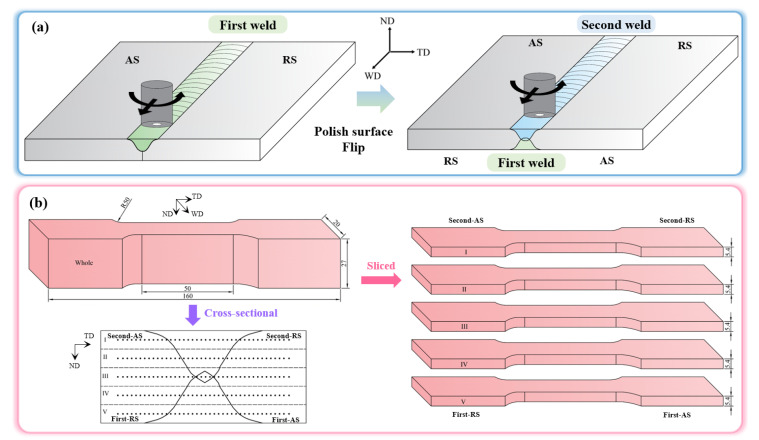
The details of the experiments. (**a**) the double-side FSW process, (**b**) the mechanical tests, including the hardness test and the tensile test (marks I–V represent the slice captured at different thickness of the joints).

The AA7A65-T7451, with a thickness of 27 mm, was used as the BM. Its optical microscope (OM) image is shown in Figure 1, and Table 1 lists its nominal chemical composition. The aluminum alloy plates were double-side butt friction stir welded at the FSW-RL31-010, as schematically illustrated in Figure 2a. The rotation speed and welding speed were set as 300 rpm and 120 mm/min, respectively. The flash and some of the arc configuration on the surface of the first weld were polished, and then the plate was flipped for the second weld. The welding direction of the first weld and second weld is the same. The two welding tools used in the current work were made up of H13 steel, and their pins were taper threaded along with triple planes. The shoulder diameter of welding tool 1 and tool 2 was 30 and 24 mm, respectively, and the pin length of them was 16 mm and 12 mm, respectively. The tool 1 was used in both the first weld and second weld of the symmetric double-side FSW joint (abbreviated as Sym joint), and the first weld and second weld of the asymmetric double-side FSW joints (abbreviated as Asy joint) were obtained by the tool 1 and tool 2, respectively.

The cross-section of joints was polished, etched with the Keller’s reagent (190 mL H_2_O+5 mL HNO_3_+3 mL HCl+2 mL HF), and observed via a Laser Scanning Confocal Microscope (LSCM, OLYMPUS, OLS4000, Tokyo, Japan) and an Optical Microscope (OM, OLYMPUS, GX71, Tokyo, Japan). The Vickers hardness test was conducted on the cross-section of the BMs and as-welded joints with a load of 200 g and a duration of 20 s using a THV-1D according to the standard (GB/T 4340.1-2009) [34]. The locations of the hardness measurement were set at the zone with a distance of 2.7, 8.1, 13.5, 18.9, and 24.3 mm from the top surface of the second weld, as shown in Figure 2b. The interval of the measuring point in the same line was 0.5 mm. Besides, the specimen for the tensile test is schematically depicted in Figure 2b. The whole joint was machined in the direction perpendicular to the welding direction (WD) and then equally divided into five slices along the normal direction (ND). The gauge scale of each slice is 50 mm × 12.5 mm × 5.4 mm. The tensile test was done at a constant rate of 1 mm/min, and the engineering strain was measured by a laser extensometer (LX500, MTS, Shanghai, China). Meanwhile, a DIC was also applied to analyze the strain distribution.

## 3. Results and Discussion

### 3.1. Weld Formation

**Figure 3:**
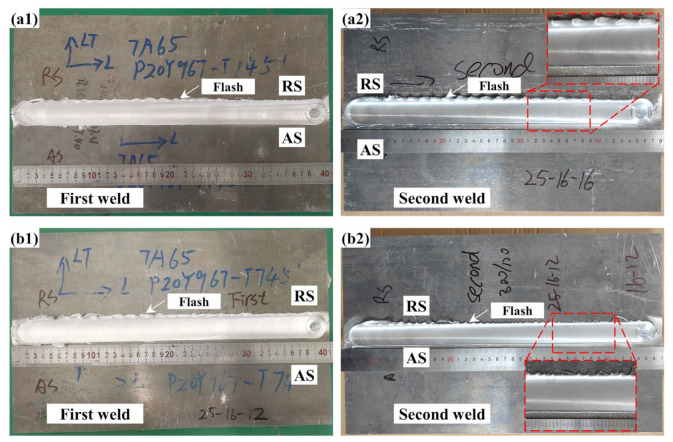
The surface morphology of the joints (**a1**,**a2**) Sym joint, (**b1**,**b2**) Asy joint.

A good weld formation is the basis of the FSW joints. Figure 3 compares the weld surface of both the Sym joint and the Asy joint to explore the effect of Asy-DS-FSW on the weld formation. The morphology of the first weld was captured immediately after the first pass was finished, and it was then polished. The typical characteristics like arc configuration and the flash can be clearly seen at both the first and second weld. The morphology in the first weld of two joints welded with the same tool is alike (Figure 3a1,b1). Further, there are obviously more flashes at the edge of the second weld in the Sym joint (Figure 3a2) than in the Asy joint (Figure 3b2). The flash is led by the squeezing out of plastic metals in the weld [35]. The volume of flashes is positively related to the degree of thermal-mechanical coupling effect because the stronger stirring action may drive more plastic materials and then force them out. Owing to the relatively smaller shoulder diameter and pin length of tool 2 than tool 1, a lower thermal-mechanical coupling effect is generated by the second pass of the Asy-DS-FSW, thus leading to a smaller flash in the Asy joint. Some researchers stated that flash is detrimental to performance because the flash may naturally provide a zone for stress concentration and corrosive medium accumulation during service [15,35]. That is, the smaller flashes generated by the Asy-DS-FSW may help to modify the weld surface forming quality and contribute to the improvement in performance.

**Figure 4:**
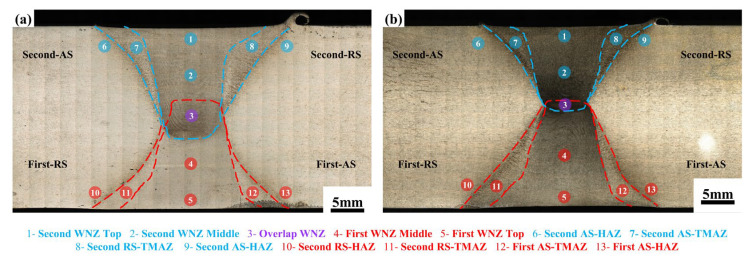
The weld cross sections of the joints (**a**) Sym joint, (**b**) Asy joint.

Figure 4 displays the macrostructure of the cross-sections of the joints. A larger flash can be seen on the RS of the second weld in the Sym joint than in the Asy joint, consistent with the observation on the weld surface in Figure 3. Both joints are completely welded, and no macroscopic welding defect can be found. Especially, the Sym joint and Asy joint exhibit no unwelded area in the WNZ which is formed in the SDS-FSW joint. The joints show an obvious dumbbell shape, with the typical weld nugget zone (WNZ), thermal-mechanically affected zone (TMAZ), heat-affected zone (HAZ) located at both the first and second weld. The formation of these zones is closely related to the heterogeneous microstructure evolution induced by the thermal-mechanical coupling effect [36]. The size of the WNZ between the first weld and second weld of the Sym joint is similar because of the same welding tool (Figure 4a). Especially in the case of Asy joints, the use of different welding tools makes two welds show different morphology (Figure 4b). The WNZ at the second weld is narrower than the first weld. The width of WNZ at Areas 1 and 2 is measured as 12.53 μm and 9.84 μm, respectively, while that at Areas 4 and 5 is 16.69 μm and 11.31 μm, respectively. A similar rule is also found in the width of TMAZ. This is because there is inevitably more friction heat and more volume of the plastic material when using the larger-scale welding tools, which then results in the larger size of the WNZ and TMAZ. Besides, the shorter pin length of tool 2 also leads to the smaller area of the overlap WNZ that experiences twice stirring (Area 3).

### 3.2. Microstructure

**Figure 5:**
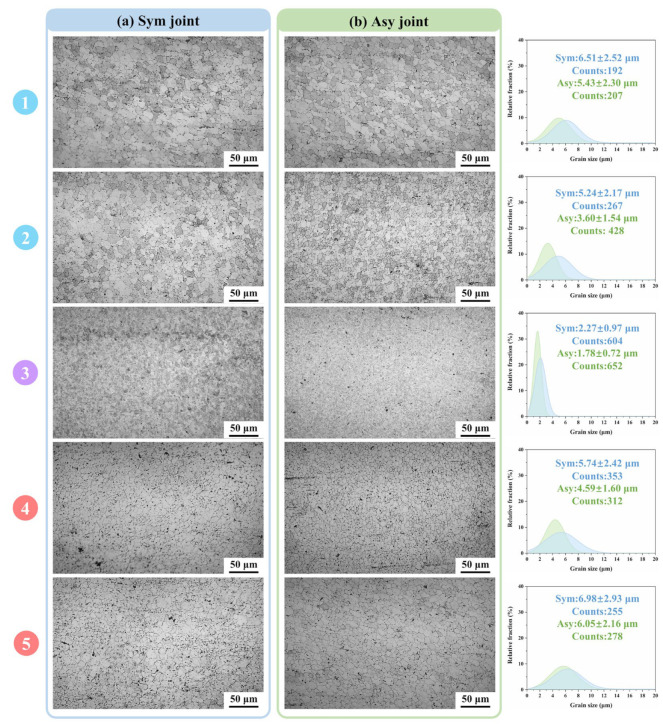
The microstructure of the WNZ (Area 1–5 in Figure 4) (**a**) Sym joint, (**b**) Asy joint.

**Figure 6:**
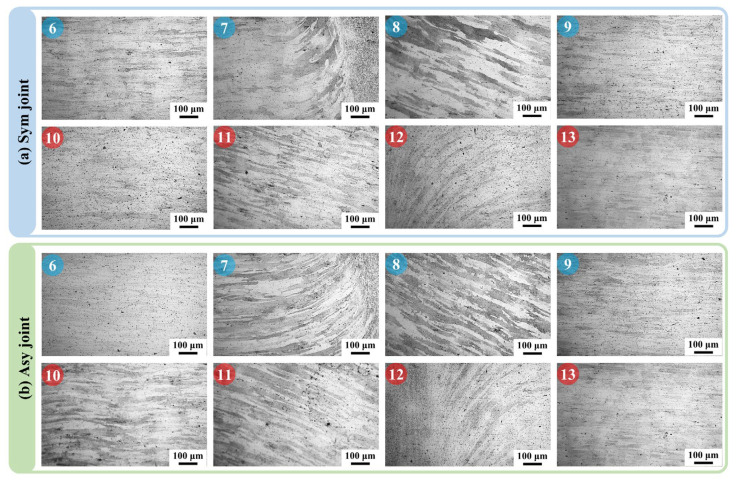
The microstructure of the TMAZ and HAZ (Area 6–13 in Figure 4).

Microstructure evolution is significant in the FSW joints. For the detailed analysis of the microstructure, Figure 5 and Figure 6 compare the OM images obtained at different zones in both Sym and Asy joints. The WNZ is composed of fine and equiaxed grains (Figure 5), which are dissimilar to the lath-shaped grains in the BM (Figure 1). This is because dynamic recrystallization occurs under the coupling effect of high temperature and severe plastic deformation. The grain size heterogeneity along the thickness direction is always an obvious issue in conventional single-pass thick-plate joints [16,29,37,38], and it becomes more complicated in the DS-FSW thick-plate joints. The corresponding grain size measurement results indicate the grain size of Area 1–5 in the Sym joints is 6.51 μm, 5.24 μm, 2.27 μm, 5.74 μm, and 6.98 μm, respectively. The grain size first decreases and then increases with increasing distance from the top surface of the second weld. The thermal cycle is believed to play the dominant role in the grain size of the WNZ [29]. The majority of the welding heat input originates from the friction heat generated via the shoulder. There is thus a higher temperature as well as a longer-high temperature duration time in the area closer to the shoulder. The grains in Areas 1 and 5 inevitably experience longer periods of grain growth than the other areas, resulting in the coarser grains there. Meanwhile, the grains in the overlap WNZ (Area 3) are refined to a greater degree because they are twice stirring and result in dynamic recrystallization. Additionally, it should be especially noted that the grains in the first weld are coarser than those in the second weld, though they were achieved under the same welding parameters. The asymmetric distribution in the grain size between the first weld and second weld demonstrates the twice grain growth in the first weld significantly facilitated by the second thermal cycle. However, this phenomenon is effectively alleviated in the Asy joint. The application of a small-size welding tool at the second weld during the Asy-DS-FSW dramatically reduces the welding heat input, lowers the peak temperature, and shortens the high-temperature duration time. As a result, the grain growth in the first weld induced by the second thermal cycle can be inhibited, making the grain size of Areas 4 and 5 reduce to 4.59 μm and 6.05 μm. Besides, the average grain size of the WNZ in the second weld (Areas 1, 2, and 3) also decreases to 5.43 μm, 3.60 μm, and 1.78 μm, respectively.

The TMAZ (Area 7, 8, 11, and 12) is the transition area between the HAZ and WNZ. Though it does not experience the mechanical stirring effect of the welding tool directly, the strong shearing force generated by the plastic material flowing in WNZ still makes the grains there elongate and bend upward (Figure 6). The bend angle of grains on the AS (Area 7 and 12) is apparently higher than that on RS (Area 8 and 11) at both the first weld and second weld. The grains at the TMAZ on AS even bend to about 90° when reaching the WNZ. Meanwhile, the heat conducted from the WNZ promotes grain growth. It is noticeable that the grain growth in TMAZ is more prominent in the Sym joint compared with the Asy joint, especially at the second weld (Areas 7 and 8). The width of the grains in TMAZ of the Sym joint is about 28 μm, while that of the Asy joint is only about 15 μm. The lower degree of grain growth in the TMAZ Asy joint should also be led by the decreased welding heat input, similar to the WNZ. The HAZ (Areas 6, 9, 10, and 13) is neighbor to the TMAZ, and the HAZ is only affected by the thermal cycle and not affected by any shear force. The grains there are thus in a similar strip shape to the BM (Figure 6). Besides, it can be seen that the grains grow along the width direction of the original strip grains owing to the thermal cycle. There is a larger width of the grains in the HAZ compared with the BM.

### 3.3. Mechanical Properties

**Figure 7:**
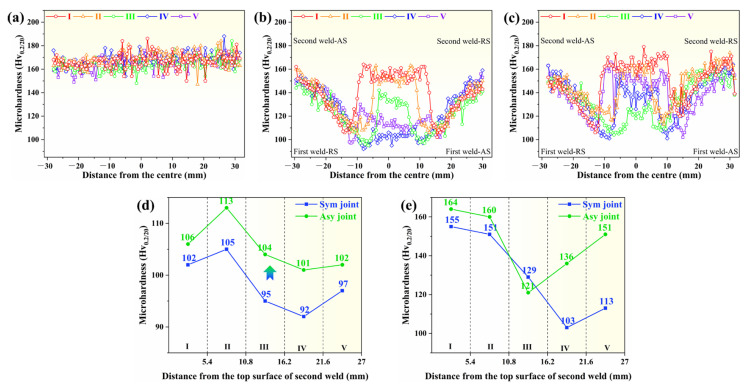
Hardness distribution of BM and joints. (**a**) BM, (**b**) Sym joint, (**c**) Asy joint, (**d**) minimum hardness of the HAZ, (**e**) average hardness of the WNZ.

The heterogeneous microstructure may result in the inhomogeneous mechanical properties of the joints. Figure 7 shows the hardness distribution profiles of the BM and joints to study the effect of Asy-DS-FSW joints on the mechanical properties heterogeneity. Homogeneous hardness distribution is exhibited in the BM, showing an average value of about 165 Hv (Figure 7a). The relatively high hardness of the AA7A65 BM should have originated from the high density of fine strengthening precipitates like G.P. zone and η’ [39]. Because of different thermal-mechanical coupling effects at different areas of the joints, all the hardness curves of slices in the joints are heterogeneous and show the typical W shape (Figure 7b,c). This is also always observed in other high-strength aluminum alloy FSW joints [6,40,41]. The significant local softening in HAZ makes it show a lower hardness than the BM and the other zones of joints. The lowest hardness at the line I–V of Sym joint is only 102 Hv_0.2/20_, 105 Hv_0.2/20_, 95 Hv_0.2/20_, 92 Hv_0.2/20_, and 97 Hv_0.2/20_, respectively (Figure 7d). It can be found that the softening in the HAZ can be suppressed via the Asy-DS-FSW, reflected by the increased lowest hardness of 106 Hv_0.2/20_, 113 Hv_0.2/20_, 104 Hv_0.2/20_, 101 Hv_0.2/20_, and 102 Hv_0.2/20_ in the HAZ of Asy joint at line I–V (Figure 7d). Besides, the hardness of WNZ in two joins all first decreases and then increases with the increasing distance from the second weld surface (Figure 7e). The average hardness of the WNZ at line I–V of the Sym joint is 155 Hv_0.2/20_, 151 Hv_0.2/20_, 129 Hv_0.2/20_, 103 Hv_0.2/20_ and 113 Hv_0.2/20_, while the corresponding value in the Asy joint is 164 Hv_0.2/20_, 160 Hv_0.2/20_, 121 Hv_0.2/20_, 136 Hv_0.2/20_, and 151 Hv_0.2/20_. The WNZ in the Asy joint is also always harder than the Sym joint, except in line III. It can also be clearly found that the measured hardness of the WNZ and HAZ at the first weld (lines IV and V) is considerably lower than that at the second weld (lines I and II) in the Sym joints, signifying the occurrence of the twice softening. However, such a large gap in the hardness between the first weld and the second weld is not observed in the Asy joint. The occurrence of the twice softening is closely related to the twice heat input during the DS-FSW. The welding heat input of the second weld may force the strengthening precipitates MgZn_2_ to coarsen or dissolve, and the grains grow again, which weakens their contribution to the strengthening [30]. There will be more obvious twice softening at the area closer to the second weld because of the more heat. Benefiting from using a combination of a large and a small welding tool, the Asy-DS-FSW produced less heat than the conventional Sym-DS-FSW applying the same large-scale welding tool. The WNZ and HAZ at the first weld of the Asy joint are thus almost unaffected, and only its WNZ at line III that is located beneath the pin experiences the twice softening, while the massive heat significantly softens the first weld of the Sym joint. A relatively smaller area of softening zone and an alleviated twice softening phenomenon eventually form in the Asy joint compared with the Sym joint.

**Figure 8:**
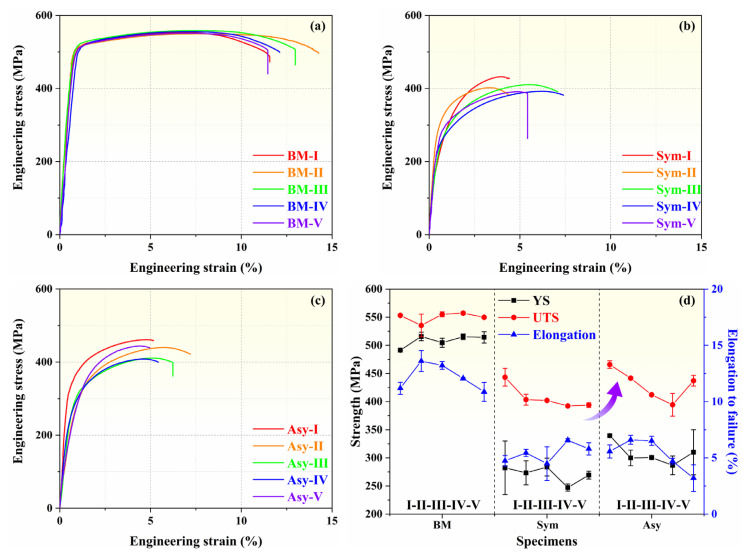
Tensile properties of BM and joints Engineering stress-engineering strain curves of BM (**a**), Sym joints (**b**), and Asy joints (**c**), the comparison of the tensile properties (**d**).

The strength and the ductility are of significance to the FSW structural components. A tensile test was carried out at the two joints to study the effect of Asy-DS-FSW on the mechanical properties of joints. Figure 8a–c displays the typical engineering stress-engineering strain curves of the slices captured in BM, Sym joint, and Asy joint, and Figure 8d summarizes the corresponding values of yield strength (YS), ultimate tensile strength (UTS), and elongation of them. The tensile properties of the five slices in the BM are almost identical, showing good homogeneity. The YS, UTS, and elongation of the slices in the BM are 508 ± 10.62 MPa, 554 ± 3.75 MPa and 12.19 ± 1.17%, respectively. The joints inevitably have worse tensile properties than the BM, and the tensile properties along the thickness direction of the joints are heterogeneous [6,36]. The UTS of the five slices in the Sym joint decreases to 443 ± 15.68 MPa, 404 ± 9.78 MPa, 402 ± 0.16 MPa, 392 ± 0.08 MPa and 394 ± 4.10 MPa, respectively, and the elongation of them is 4.74 ± 0.47%, 5.03 ± 0.91%, 6.74 ± 0.48%, 7.03 ± 0.51% and 5.81 ± 0.55%. It is worth noting that the strength of the first weld (IVand V) is lower than the second weld (I and II), consistent with the hardness distribution, emphasizing the significant twice softening phenomenon. In comparison, the UTS of the I–V slices can reach 466 ± 6.65 MPa, 442 ± 2.42 MPa, 412 ± 1.62 MPa, 395 ± 20.34 MPa, and 437 ± 9.53 MPa, respectively. The Asy joints exhibit an enhancement in the strength of the slices. Their joining efficiencies are 5.1%, 9.5%, 2.5%, 0.5%, and 11% higher than those in the Sym joint, respectively. This should also profit from the small welding tool in the second pass and the resultant weakened thermal cycle. Meanwhile, the elongation of them is 5.58 ± 0.59%, 7.04 ± 0.22%, 6.52 ± 0.40%, 5.16 ± 0.37%, and 3.65 ± 1.82%, respectively. It can be seen that the elongation of the Asy joint was higher than that of the Sym joints at the second weld but lower than that of the Sym joint at the first weld.

**Figure 9:**
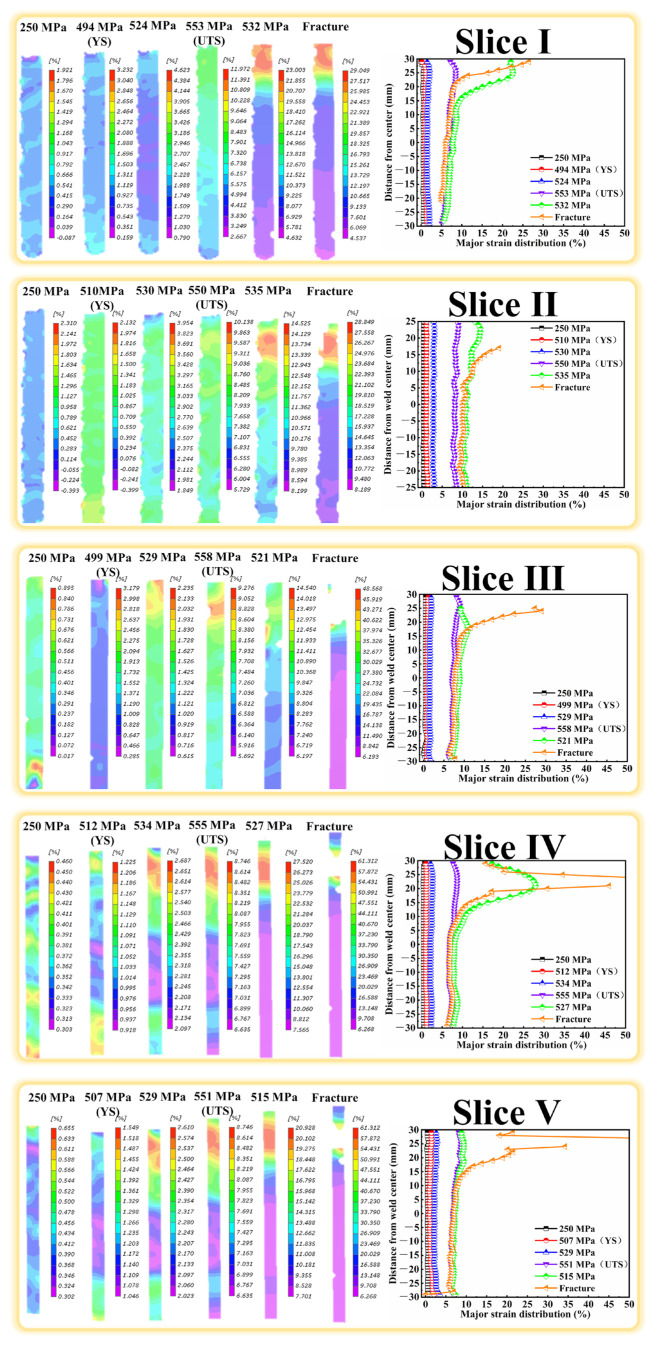
The strain distribution of the slices in BM.

**Figure 10:**
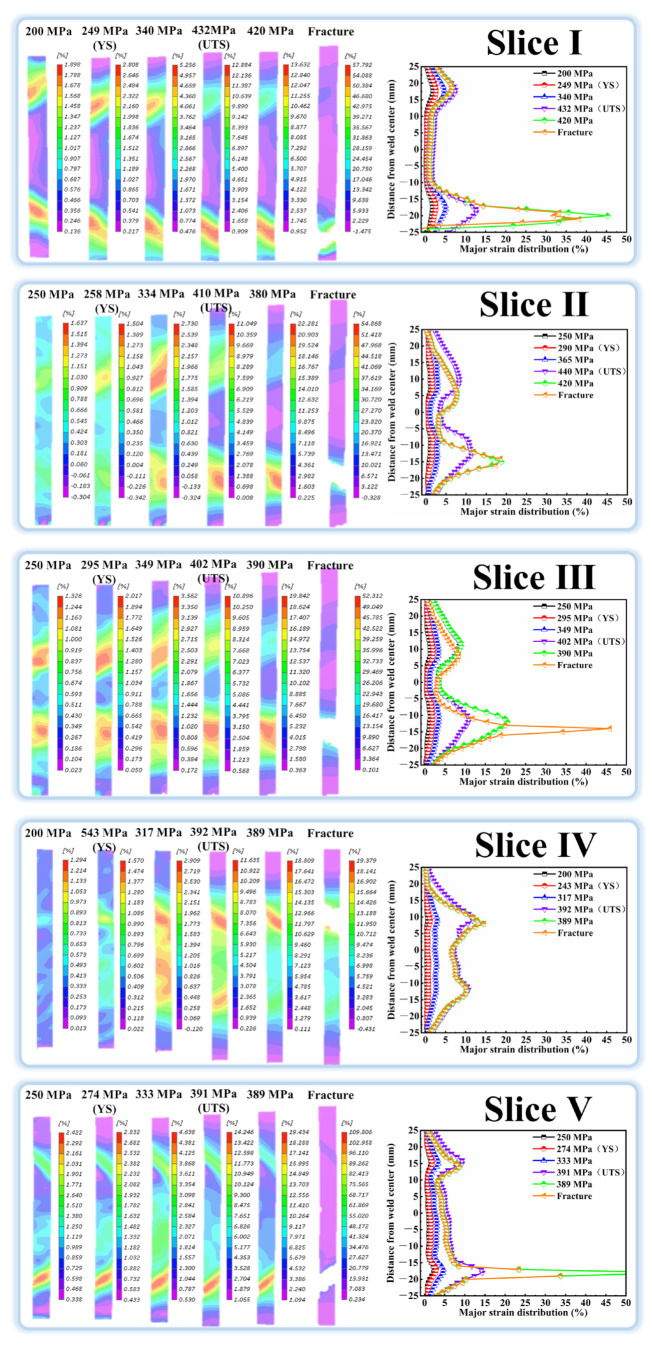
The strain distribution of the slices in Sym joint.

**Figure 11:**
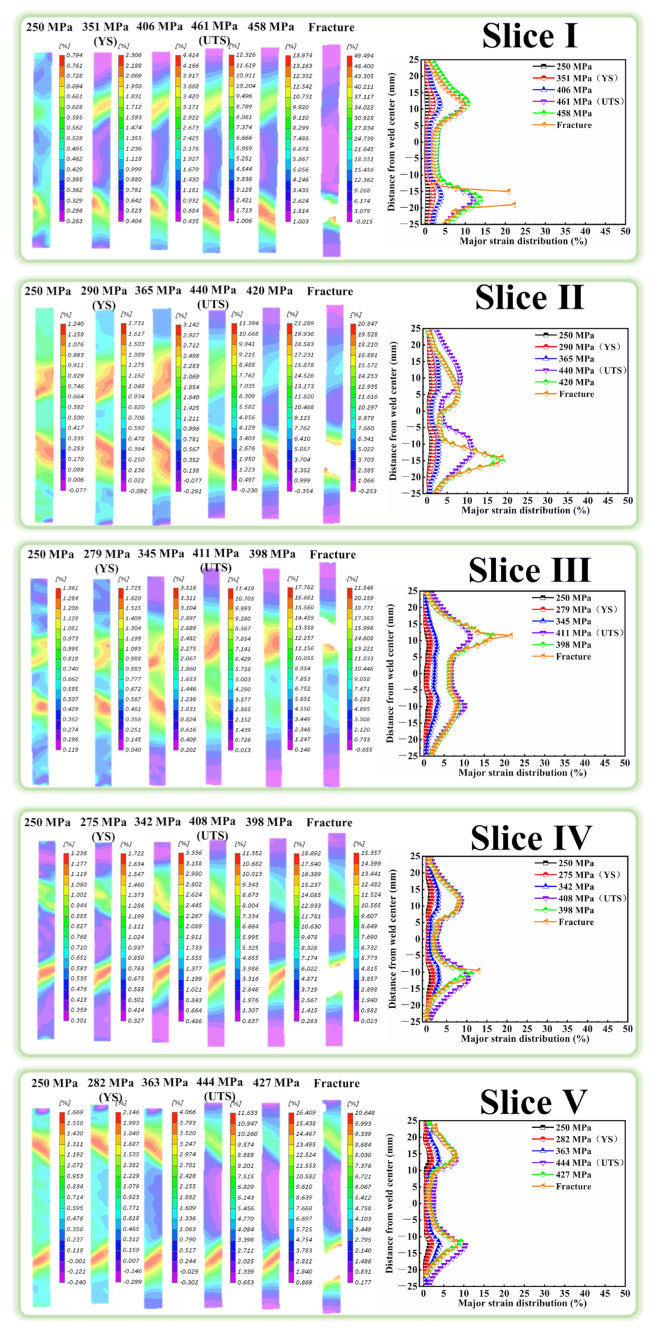
The strain distribution of slices in the Asy joint.

Owing to the macroscopic microstructure heterogeneity, the FSW joints will exhibit uneven strain distribution, which significantly affects the performance of the joints. DIC was thus applied during the tensile test to characterize the strain distribution. Figure 9, Figure 10 and Figure 11 show the cross-sectional strain distribution of the slices in BM and joints at six stages (the elastic deformation, YS, strain hardening, UTS, prior to fracture and fracture) under the monotonic load. It can be clearly seen in Figure 9 that the deformation of all the slices in BM is uniform before the stress reaches the UTS. The plastic deformation begins to localize at the edge of the gauge during the necking stage, finally leading to the fracture there. In the case of the slices in joints Figure 10 and Figure 11, it is evident that their strain distribution curves always show the typical “M” shape, contrary to hardness distribution curves. The strain localization is prone to occur at the HAZ with the lowest hardness, and the deformation in the other zones is relatively low. The strain localization can even initiate in the HAZ at the elastic deformation stage. The maximum strain in HAZ then continuously intensifies with the gradually increasing stress. The slices of joints eventually fracture in the HAZ, showing the maximum strain.

**Figure 12:**
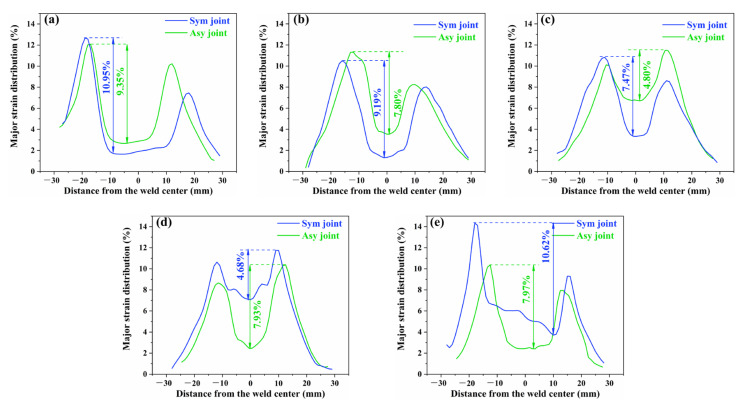
Comparison in the strain distribution between the Sym joint and Asy joint at the respective UTS (**a**) slice I, (**b**) slice II, (**c**) slice III, (**d**) slice IV, (**e**) slice V.

The strain localization always limits the strength and plastic deformation capacity of the global joint and promotes premature fracture of the joints [42], which is significant to the service reliability of joints. Figure 12 compares the strain distribution curves of the slices between the Sym joint and Asy joint at the respective UTS to further analyze the role of Asy-DS-FSW on strain localization. The bulge of strain can visually reflect the degree of strain localization. The Sym joint always exhibits a more prominent bulge of strain than the Asy joint, demonstrating the more severe strain localization there. This phenomenon is mainly attributed to the much softer HAZ and the larger gap in the hardness between the HAZ and WNZ in the Sym joint (Figure 7) compared with the Asy joint. The deteriorative hardness homogeneity is harmful to the deformation coordination between different zones, which strongly aggravates the strain localization in the Sym joint. The performance of the Sym joint thus inevitably degrades. Whereas the strain localization of the slices in the Asy joint is eased owing to the more homogeneous microstructure and hardness distribution generated by the typical Asy-DS-FSW strategy.

### 3.4. Fracture Behavior

**Figure 13:**
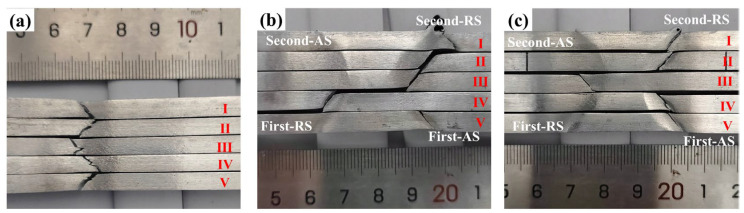
Macroscopic fracture morphology of BM and joints. (**a**) BM, (**b**) Sym joint, (**c**) Asy joint. (marks I–V represent the slice captured at different thickness of the joints).

**Figure 14:**
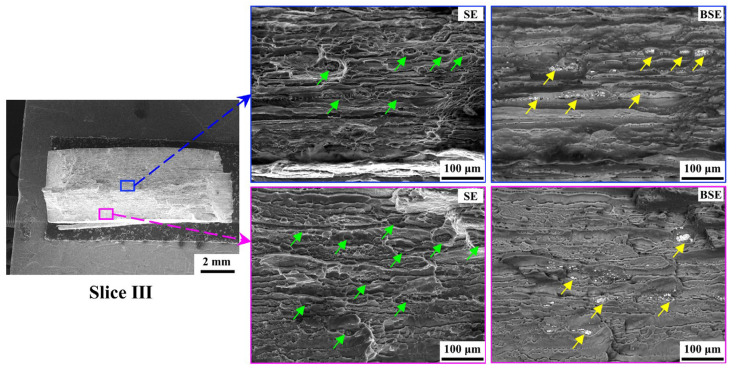
Fracture surface of slice III in BM (green arrows and yellow arrows represent the dimples and the broken particles, respectively).

The fracture morphology was observed macroscopically and microscopically via the camera and SEM in order to understand the effect of Asy-DS-FSW on fracture behaviors. Figure 13 illustrates the macroscopic fracture morphology of the BM and the joints. The fracture surface of the BM is 45° to the loading direction, showing the typical characteristic of the shear fracture (Figure 13a). Meanwhile, significant necking can be observed in the BM, well consistent with its high elongation in Figure 8. A similar 45° shear fracture also occurs at the HAZ in both the Sym joint and the Asy joint, but the necking of the joints seems relatively weak, aligned with their deteriorated elongation. The fracture behavior of the BM and joints was further analyzed according to the characterization of their fracture surfaces. Figure 14 shows the typical SEM images of the fracture surface of the BM captured in slice III. It can be clearly seen from the secondary electron (SE) images that there are lots of dimples along with the tearing ridges in the BM (indicated with green arrows). The corresponding backscatter electron (BSE) images show the broken particles lying beneath the dimples (indicated with yellow arrows). These particles should be the Fe-rich intermetallics formed during the casting of the BM [43]. These characteristics are the symbol of the microvoid coalescence ductile fracture. The deformation of the particles is not always in sync with the surrounding Al matrix because the modulus of the particles is different from the surrounding Al matrix. The boundaries between the particles and the Al matrix are thus prone to microvoid formation. The microvoids then coalesce together, eventually generating the main crack along the 45° of the BM.

**Figure 15:**
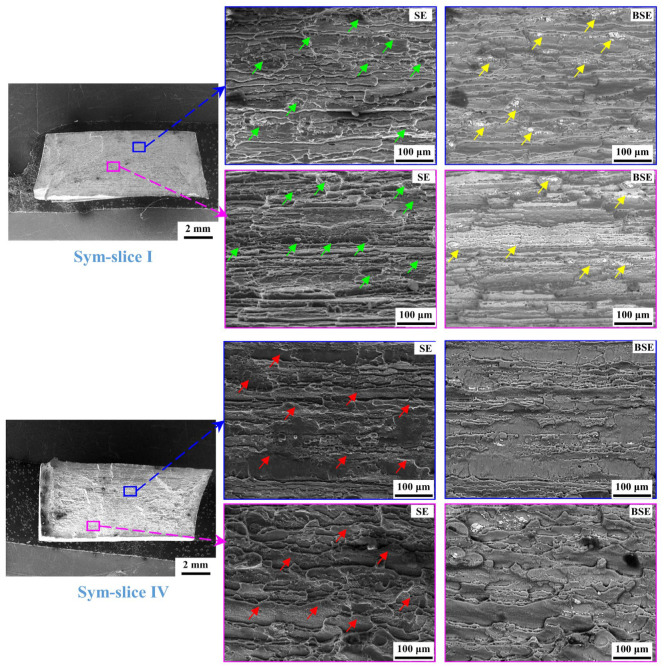
Fracture surface of slices I and IV in Sym joint (green arrows, yellow arrows, and red arrows represent the dimples, the broken particles, and quasi-cleavage steps, respectively).

Figure 15 depicts the fracture surface of the slice I and IV of the Sym joint. Its fracture morphology is greatly different from the BM. The number of dimples there is less and the dimples are smaller and shallower compared with the BM. This suggests the dimples in the Sym joint cannot fully grow to a larger size as the BM after they nucleate. This should be owing to the aggravated deformation dissonance between the particles and the surrounding Al matrix in the softened HAZ. Especially this phenomenon is more severe in slice IV than in slice I. As seen in the fracture surface of slice IV, there are little dimples and the particles beneath dimples, while numerous quasi-cleavage steps can be seen (indicated with red arrows). These features indicate the relatively weak ability to bear the tensile stress for slice IV. Noteworthy, though the elongation of slice IV is higher than that of slice I in the Sym joint (Figure 8), the strain distribution obtained by DIC (Figure 10 and Figure 12) suggests a higher local major strain in the HAZ of slice I than the slice IV, i.e., the better deformation ability in slice I. The transition in the fracture surface from slice I to slice IV further confirms the detrimental role of the twice softening induced by the twice thermal cycle.

**Figure 16:**
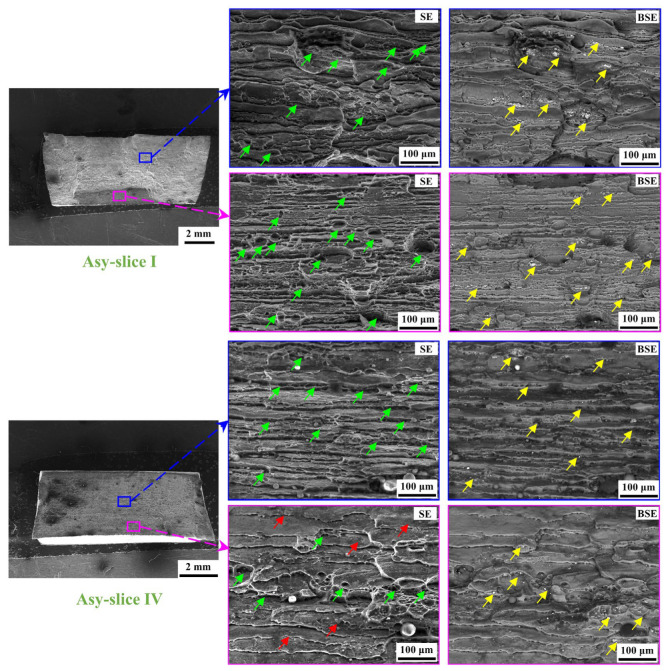
Fracture surface of slices I and IV in Asy joint (green arrows, yellow arrows, and red arrows represent the dimples, the broken particles, and quasi-cleavage steps, respectively).

However, slices I and IV in the Asy joint exhibit similar fracture morphology, as shown in Figure 16. The dimples, along with the tearing ridges, can be observed. There are more dimples with larger dimensions in the Asy joint than in the Sym joint. The growth of dimples may help delay crack propagation and energy absorption, thereby improving damage tolerance. Besides, the number of quasi-cleavage steps decreases, indicating the reduced tendency of the brittle fracture. These phenomena should be led by the alleviated twice softening achieved by the Asy-DS-FSW.

## 4. Conclusions

In this research, the Asy-DS-FSW was proposed, and it was carried out at the 27 mm thick AA7A65-T7451. The weld formation microstructure and the mechanical properties of the conventional Sym-DS-FSW and proposed Asy-DS-FSW joints were investigated and compared. The following conclusions are drawn:

(1) The Asy-DS-FSW helps optimize the surface forming quality of the weld because the application of the smaller size welding tool in the second weld can hinder the generation of the flash.

(2) Profiting from the less heat generated via the smaller size welding tool, not only the grains in the second weld of the Asy-DS-FSW joint are refined, but also the grain growth in its first weld led by the second thermal cycle is effectively inhibited.

(3) The hardness of the Asy-DS-FSW joints is higher and more uniformly distributed than the conventional Sym-DS-FSW joints because of the alleviated twice softening, which eases the strain localization and improves the strength of joints. The UTS of the slice I, II, III, IV, and V in the Asy-DS-FSW joints can reach 84.67%, 80.29%, 74.91%, 71.69%, and 79.45% of the BM, 5.1%, 9.5%, 2.5%, 0.5%, and 11% higher than those in the Sym-DS-FSW joint, respectively.

(4) The proposed Asy-DS-FSW provides a viable and advantageous method for high-quality joining high-strength aluminum alloy thick-plates in aircraft manufacturing.

## 5. Outlook

The current work proposes an Asy-DS-FSW method for thick-plate Al-Zn-Mg-Cu alloys. The work preliminarily confirms this method shows comprehensive advantages in practical applications, including lower demand on tools and equipment, lower difficulty but higher quality of the weld formation, less welding heat input, better microstructure homogeneity, and consequent improved joining efficiency compared with single-side FSW, BB-FSW, SDS-FSW, and the conventional Sym-DS-FSW. The welding parameters, like rotation speed and welding speed, etc., dominate the welding heat input and the consequent microstructure, like grains and precipitates of the joints. Besides, the choice of welding tool may also be crucial, especially the geometry and size of the shoulder and the pin. This provides a large processing space for the weld formation optimization, microstructure modification, and performance improvement of thick-plate high-strength aluminum alloys or other alloys. Future study on the processing-microstructure-properties relationship is required for a more comprehensive understanding of this promising technology.

## Figures and Tables

**Table 1:** Nominal chemical composition of AA7A65 (wt%) [33].

Al	Zn	Mg	Cu	Fe	Si	Mn	Ti	Zr	Cr
Bal.	7.70	1.65	1.00	0.08	0.06	0.04	0.06	0.10	0.04

## Data Availability

The raw/processed data required to reproduce these findings would remain confidential and would not be shared at this time because the data also forms part of an ongoing study.

## References

[1] Dursun T., Soutis C. (2014). Recent developments in advanced aircraft aluminium alloys. Mater. Des..

[2] Guo X., Li H., Xue P., Pan Z., Xu R., Ni D., Ma Z. (2023). Microstructure and mechanical properties of 600 MPa grade ultra-high strength aluminum alloy fabricated by wire-arc additive manufacturing. J. Mater. Sci. Technol..

[3] Liu P., Liu F.C., Wang Y.D., Zhang Z., Xue P., Wu L.H., Zhang H., Ni D.R., Xiao B.L., Ma Z.Y. (2024). Additive manufacturing of commercial Al–Zn–Mg–Cu aluminum alloys with mechanical properties comparable to extruded counterparts. Mater. Sci. Eng. A.

[4] Starke E.A., Staley J.T. (1996). Application of modern aluminum alloys to aircraft. Prog. Aerosp. Sci..

[5] Liu F.C., Feng A.H., Pei X., Hovanski Y., Mishra R.S., Ma Z.Y. (2024). Friction stir based welding, processing, extrusion and additive manufacturing. Prog. Mater. Sci..

[6] Xu W.F., Wang H., Lu H.J., Liu Y.L., Dong J.H. (2021). Effect of thermal exposure on microstructure and mechanical properties of friction stir welding 7B50-T7451 aluminium alloy thick plate joint. J. Mater. Res. Technol..

[7] Xu W.F., Luo Y.X., Zhang W., Fu M.W. (2018). Comparative study on local and global mechanical properties of bobbin tool and conventional friction stir welded 7085-T7452 aluminum thick plate. J. Mater. Sci. Technol..

[8] Zhang Z., Xiao B.L., Ma Z.Y. (2014). Hardness recovery mechanism in the heat-affected zone during long-term natural aging and its influence on the mechanical properties and fracture behavior of friction stir welded 2024Al-T351 joints. Acta Mater..

[9] Ma Z.Y., Feng A.H., Chen D.L., Shen J. (2017). Recent advances in friction stir welding/processing of aluminum alloys: Microstructural evolution and mechanical properties. Crit. Rev. Solid State Mater. Sci..

[10] Feng A.H., Chen D.L., Ma Z.Y. (2010). Microstructure and Low-Cycle Fatigue of a Friction-Stir-Welded 6061 Aluminum Alloy. Metall. Mater. Trans. A.

[11] Feng A.H., Chen D.L., Ma Z.Y. (2010). Microstructure and Cyclic Deformation Behavior of a Friction-Stir-Welded 7075 Al Alloy. Metall. Mater. Trans. A.

[12] Heidarzadeh A., Mironov S., Kaibyshev R., Çam G., Simar A., Gerlich A., Khodabakhshi F., Mostafaei A., Field D.P., Robson J.D. (2021). Friction stir welding/processing of metals and alloys: A comprehensive review on microstructural evolution. Prog. Mater. Sci..

[13] Xu W.F., Lu H.J., Li X.H., Wang M., Ma J., Luo Y.X. (2021). Microstructure evolution and stress corrosion cracking sensitivity of friction stir welded high strength AA7085 joint. Mater. Des..

[14] Mao Y.Q., Ke L.M., Liu F.C., Chen Y.H. (2017). Formation mechanism of weld loose defect in friction stir welding thick plates of aluminum alloy. Acta Aeronaut. Astronaut. Sin..

[15] Meng X.C., Huang Y.X., Cao J., Shen J.J., dos Santos J.F. (2021). Recent progress on control strategies for inherent issues in friction stir welding. Prog. Mater. Sci..

[16] Shi L., Dai X., Tian C.Y., Wu C.S. (2022). Effect of splat cooling on microstructures and mechanical properties of friction stir welded 2195 Al–Li alloy. Mater. Sci. Eng. A.

[17] Singh V.P., Patel S.K., Kuriachen B. (2021). Mechanical and microstructural properties evolutions of various alloys welded through cooling assisted friction-stir welding: A review. Intermetallics.

[18] Sinhmar S., Dwivedi D.K. (2017). Enhancement of mechanical properties and corrosion resistance of friction stir welded joint of AA2014 using water cooling. Mater. Sci. Eng. A.

[19] Xu W.F., Liu J.H., Chen D.L., Luan G.H., Yao J.S. (2012). Improvements of strength and ductility in aluminum alloy joints via rapid cooling during friction stir welding. Mater. Sci. Eng. A.

[20] Wang J., Yang K., Zhang Y.A., Lu Y.-L., Bai Z.-H., Li X.-C. (2021). Investigation on variations of microstructures and mechanical properties along thickness direction of friction stir processed AA2014 aluminum alloy via ultra-rapid cooling. Mater. Charact..

[21] Dong J.L., Zhang D.T., Zhang W.W., Zhang W., Qiu C. (2019). Microstructure and properties of underwater friction stir-welded 7003-T4/6060-T4 aluminum alloys. J. Mater. Sci..

[22] Li X., Zhang Z., Peng Y., Yan D., Tan Z., Zhou Q., Wang K., Zhou M. (2022). Microstructure and mechanical properties of underwater friction stir welding of CNT/Al-Cu-Mg composites. J. Mater. Res. Technol..

[23] Zhao Y., Wang Q., Chen H., Yan K. (2014). Microstructure and mechanical properties of spray formed 7055 aluminum alloy by underwater friction stir welding. Mater. Des..

[24] Xu W.F., Luo Y., Fu M.W. (2018). Microstructure evolution in the conventional single side and bobbin tool friction stir welding of thick rolled 7085-T7452 aluminum alloy. Mater. Charact..

[25] Chu Q., Li W.Y., Wu D., Liu X.C., Hao S.J., Zou Y.F., Yang X.W., Vairis A. (2021). In-depth understanding of material flow behavior and refinement mechanism during bobbin tool friction stir welding. Int. J. Mach. Tools Manuf..

[26] Wu D., Li W.Y., Gao Y.J., Yang J., Su Y., Wen Q., Vairis A. (2020). Effect of an improved pin design on weld formability and mechanical properties of adjustable-gap bobbin-tool friction stir welded Al-Cu aluminum alloy joints. J. Manuf. Processes.

[27] Zou Y.F., Li W.Y., Tang Y.S., Su Y., Yang X.W., Wu D., Wang W.B. (2023). A comparative study of microstructure and mechanical properties of conventional and synergistic double-sided FSW joints of 6061 zxaluminium alloy. Sci. Technol. Weld. Join..

[28] Tang Y., Li W., Zou Y., Wang W., Xu Y., Vairis A., Çam G. (2024). Effects of tool rotation direction on microstructure and mechanical properties of 6061 aluminum alloy joints by the synergistically double-sided friction stir welding. J. Manuf. Processes.

[29] Yang C., Zhang J.F., Ma G.N., Wu L.H., Zhang X.M., He G.Z., Xue P., Ni D.R., Xiao B.L., Wang K.S. (2020). Microstructure and mechanical properties of double-side friction stir welded 6082Al ultra-thick plates. J. Mater. Sci. Technol..

[30] Li Y., He C.S., Wei J.X., Zhang Z.Q., Qin G.W., Zhao X. (2021). Correlation of local microstructures and mechanical properties of Al–Zn–Mg–Cu alloy build fabricated via underwater friction stir additive manufacturing. Mater. Sci. Eng. A.

[31] Zhou Y., Lin X., Kang N., Huang W., Wang Z. (2021). Mechanical properties and precipitation behavior of the heat-treated wire + arc additively manufactured 2219 aluminum alloy. Mater. Charact..

[32] Maia P.P.N., Miná É.M., Dalpiaz G., Marinho R.R., Paes M.T.P., Motta M.F., de Miranda H.C., Silva C.C. (2023). Microstructural characterisation and micromechanical investigation of the heat-affected zone in multi-pass welding of 9Ni steel pipes. J. Mater. Res. Technol..

[33] Li C., Chen Z., Zhang X.-Q., Xiao W.-L., Ma Y. (2022). Hot deformation behavior of high Zn-containing 7A65 Al alloy. Rare Met..

[34] (2009). Metallic Materials Vickers Hardness Test Part 1: Test Method, Standardization Administration of the People’s Republic of China.

[35] Zeng K.-W., Su Z.-M., Luo S.-M., Lin P.-C., Dong M.-T., Tang T., Huang B. (2013). Removing approach for flashes of friction stir spot welds. J. Mater. Processes Technol..

[36] Xu W.F., Wang H., Luo Y.X., Li W.J., Fu M.W. (2018). Mechanical behavior of 7085-T7452 aluminum alloy thick plate joint produced by double-sided friction stir welding: Effect of welding parameters and strain rates. J. Manuf. Processes.

[37] Xu W.F., Liu J.H., Luan G., Dong C. (2009). Temperature evolution, microstructure and mechanical properties of friction stir welded thick 2219-O aluminum alloy joints. Mater. Des..

[38] Wang H., Xu W.F., Lu H.J., Liu Y.L. (2022). Effect of microstructure inhomogeneity on creep behavior of friction stir welding 7B50-T7451 aluminum alloy thick plate joint. Mater. Charact..

[39] Xiao W.L., Zang C.Y., Guo J.T., Feng J.W., Ma C.L. (2024). Two-stage aging process of 7A65 aluminum alloy thick plate based on in situ resistance method. Acta Metall. Sin..

[40] Gupta S., Haridas R.S., Agrawal P., Mishra R.S., Doherty K.J. (2022). Influence of welding parameters on mechanical, microstructure, and corrosion behavior of friction stir welded Al 7017 alloy. Mater. Sci. Eng. A.

[41] Xu W.F., Wu X.K., Ma J., Lu H.J., Luo Y.X. (2019). Abnormal fracture of 7085 high strength aluminum alloy thick plate joint via friction stir welding. J. Mater. Res. Technol..

[42] Dai W., Guo W., Li Q., Xiao J., Li W., Zhang H. (2024). Homogenization of local microstructure and mechanical properties in friction stir welded Al-Cu alloy joint achieved through laser shock peening. J. Mater. Processes Technol..

[43] Bertoncello J.C.B., Manhabosco S.M., Dick L.F.P. (2015). Corrosion study of the friction stir lap joint of AA7050-T76511 on AA2024-T3 using the scanning vibrating electrode technique. Corros. Sci..

